# Time-dependent apoptosis induction after spontaneous-breathing or ventilation-analogue experimental mechanostimulation of monolayer lung cell cultures

**DOI:** 10.1186/cc13469

**Published:** 2014-03-17

**Authors:** S Meyer, S Schumann, J Guttmann, K Gamerdinger

**Affiliations:** 1 University Medical Center Freiburg, Germany

## Introduction

The cyclic strain of lung tissue during mechanical ventilation compared with spontaneous breathing is associated with a largely increased mechanical load due to larger pressure amplitudes and higher acceleration forces. This additional load may change tissue mechanics and may lead to tissue damage. Although experimental mechanostimulation in vitro is a widely used method to analyse mechanical load on cells or tissue, in vitro experiments with ventilation-analogue stimulation are missing. In this work we compare for the first time the changes of monolayer lung cells after ventilation-analogue stimulation with spontaneous-breathing analogue mechanostimulation. The aim of the study was to show time- dependent differences in the induction of apoptosis due to mechanical overload, by fluorescence microscopic observations.

## Methods

Alveolar epithelial cells (A549) were grown on RGD-coated, highly flexible polydimethyl siloxane membranes. After becoming approximately 100% confluent, cells were stained with Hoechst 33342/TMRE/caspase-3 and caspase-7/PI and continuously observed by fluorescence microscopy. The cells were stimulated either with ventilation-analogue or spontaneous-breathing analogue profile [1]. For both settings the frequency was 0.25 Hz, the surface increase 20% and the cell monolayers were stimulated over a time period of 2 hours in the bioreactor [2].

## Results

The analysis of fluorescence microscopic images showed the first evidence of apoptosis induction after 40 minutes of ventilation-analogue stimulation. In contrast, apoptosis induction after spontaneous-breathing analogue stimulation occurred after 90 minutes.

## Conclusion

The observation of cells during stimulation with continuous fluorescence microscopic imaging allowed us to analyse the time- dependent induction of apoptosis under the aspects of different stimuli. We could prove our hypothesis that ventilation-analogue stimulation was worse for the cellular viability then spontaneous- breathing analogue stimulation. In future, the direct optical tracking of cell damage should allow one to analyse other stimulation profiles as well, and thereby to improve the stimulation profiles and ultimately the ventilation profile as well.
